# Identifying Quantitative Trait Loci Affecting Resistance to Congenital Hypothyroidism in 129*^+Ter^*/SvJcl Strain Mice

**DOI:** 10.1371/journal.pone.0031035

**Published:** 2012-01-27

**Authors:** Yayoi Hosoda, Nobuya Sasaki, Yayoi Kameda, Daisuke Torigoe, Takashi Agui

**Affiliations:** Laboratory of Laboratory Animal Science and Medicine, Department of Disease Control, Graduate School of Veterinary Medicine, Hokkaido University, Sapporo, Japan; Emory University School of Medicine, United States of America

## Abstract

Tyrosylprotein sulfotransferase 2 (TPST2) is one of the enzymes responsible for tyrosine O-sulfation and catalyzes the sulfation of the specific tyrosine residue of thyroid stimulating hormone receptor (TSHR). Since this modification is indispensable for the activation of TSH signaling, a non-functional TPST2 mutation (*Tpst2^grt^*) in DW/J-*grt* mice leads to congenital hypothyroidism (CH) characterized by severe thyroid hypoplasia and dwarfism related to TSH hyporesponsiveness. Previous studies indicated that the genetic background of the 129*^+Ter^*/SvJcl (129) mouse strain ameliorates *Tpst2^grt^*-induced CH. To identify loci responsible for CH resistance in 129 mice, we performed quantitative trait locus (QTL) analysis using backcross progenies from susceptible DW/J and resistant 129 mice. We used the first principal component calculated from body weights at 5, 8 and 10 weeks as an indicator of CH, and QTL analysis mapped a major QTL showing a highly significant linkage to the distal portion of chromosome (Chr) 2; between *D2Mit62* and *D2Mit304*, particularly close to *D2Mit255*. In addition, two male-specific QTLs showing statistically suggestive linkage were also detected on Chrs 4 and 18, respectively. All QTL alleles derived from the 129 strain increased resistance to growth retardation. There was also a positive correlation between recovery from thyroid hypoplasia and the presence of the 129 allele at *D2Mit255* in male progenies. These results suggested that the major QTL on Chr 2 is involved in thyroid development. Moreover, since DW/J congenic strain mice carrying both a *Tpst2^grt^* mutation and 129 alleles in the major QTL show resistance to dwarfism and thyroid hypoplasia, we confirmed the presence of the resistant gene in this region, and that it is involved in thyroid development. Further genetical analysis should lead to identification of genes for CH tolerance and, from a better understanding of thyroid organogenesis and function, the subsequent development of new treatments for thyroid disorders.

## Introduction

Congenital hypothyroidism (CH) is one of the most frequent endocrinological disorders. It is caused by insufficient production of thyroid hormone by the thyroid gland, and about 1∶3000–4000 newborns are affected by CH. If untreated, they suffer from irreversible growth delay and mental retardation. In most cases (80–90%), CH is caused by thyroid dysgenesis (TD) characterized by abnormal thyroid gland development, such as ectopic thyroid gland, athyreosis and thyroid hypoplasia [1], [2]. Both environmental and genetic factors affect the development of TD [3]–[5]. Molecular genetic analyses have identified some TD susceptible genes in humans; thyroid stimulating hormone receptor (*TSHR*), thyroid transcription factor 1 (*TTF1*), *TTF2*, paired box gene 8 (*PAX8*), *NKX2.5* and *HHEX* [1], [6], [7]. Studies of the spontaneous mutation and targeted-disruption of the above genes in mice have also provided insights into the molecular mechanisms of thyroid organogenesis and thereby formed the basis for molecular genetic studies in human patients affected by TD [1], [8]. Numerous studies have used a candidate gene approach to identify genes involved in common forms of TD. However, most forms of TD appear sporadically, and no significant linkage between the TD phenotype and haplotypes surrounding T*SHR*, *TTF1*, *TTF2* and *PAX8* locus have been reported [9]. Moreover, the incomplete penetrance and the variable expression observed in familial cases of hypothyroidism demonstrate that TD-associated CH is a genetically heterogeneous disease [9], [10]. Thus, there is little information available on the genetic factors involved in thyroid disease.

DW/J-*grt* is a mouse model for TD-associated CH that is characterized by autosomal recessive growth retardation, significantly lowered T3 and T4 levels, and severe thyroid hypoplasia related to TSH hyporesponsiveness [11]. Previously, we identified a point mutation (*Tpst2^grt^*) in the tyrosylprotein sulfotransferase 2 (*Tpst2*) gene causing a decrease in enzymatic activity [12]. TPST2 is one of the enzymes responsible for tyrosine O-sulfation and catalyzes the sulfation of tyrosine 385 of TSHR [13]. Since this modification is indispensable for the activation of TSH signaling [12]–[14] and, furthermore, since signal transduction via TSHR is prerequisite for the development and function of thyroid gland [15], [16], DW/J-*grt* mice develop CH [12]. Previously, to examine the effects of genetic background on hypothyroidism, we produced congenic strains carrying this *Tpst2^grt^* mutation on the genetic background of standard strains, C57BL6/J (B6) and 129*^+Ter^*/SvJcl (129), and analyzed growth rate and thyroid function. The B6 congenic mice show a severe hypothyroid phenotype similar to the DW/J (DW) strain. In contrast, and interestingly, the 129 congenic mice exhibit normal growth and thyroid function. This result suggests that 129 strain have resistant gene(s) against CH [17]. In order to identify the resistant gene(s) against *Tpst2^grt^*-induced CH, we carried out quantitative trait locus (QTL) analysis using backcross progenies from susceptible DW and resistant 129 mice. Because the tolerance to growth retardation in 129 strain is caused by the normal thyroid development and function in spite of TSH hyporesponsiveness [17], body weight is expected to reflect the severity of CH. We used the first principal component of body weights at 5, 8 and 10 weeks age, calculated by principal component analysis, as an indicator of CH. QTL analysis mapped a major QTL showing a highly significant linkage to the distal portion of chromosome (Chr) 2; between *D2Mit62* and *D2Mit304*, particularly close to *D2Mit255*. In addition, a further two QTLs showing suggestive statistical linkages were detected on Chrs 4 and 18, respectively, in the male progenies. High percentages of the variance (63–69%) at *D2Mit255* also supported the validity of the QTL on Chr 2 as a powerful modifier for growth delay by *Tpst2^grt^*. Statistically significant correlations between body weight and thyroid index for both sexes demonstrated that the growth retardation in backcross progeny is related to thyroid hypoplasia. Also, a significant difference in male thyroid index between genotypes at *D2Mit255* suggested the major tolerant gene on Chr 2 is involved in thyroid development. Finally, we generated DW congenic strain mice carrying both a *Tpst2^grt^* mutation and 129 alleles in the major QTL, and confirmed the recovery from both growth retardation and thyroid hypoplasia.

## Materials and Methods

### Ethical Statement

All research and experimental protocols were approved by the Regulation for the Care and Use of Laboratory Animals, Hokkaido University (approval ID: No. 110226), and performed under the guidance of the Institute for Laboratory Animal Research (ILAR). All animals were housed in a facility approved by the American Association for Accreditation of Laboratory Animal Care (AAALAC) International.

### Animals

129 mice were purchased from CLEA Japan (129*^+Ter^*/SvJcl; Tokyo, Japan). Backcross progenies were obtained by mating DW female mice heterozygous for *Tpst2^grt^* to DW129-F1 hybrid male mice heterozygous for *Tpst2^grt^*. Backcross mice homozygous for *Tpst2^grt^* (BC-*Tpst2^grt^*) were born in accordance with Mendelian inheritance and the equal sex ratio. Backcross progenies with homozygotic wild-type alleles for *Tpst2* gene (BC-WT) were used as littermate controls. Genotyping of the *Tpst2^grt^* allele was performed as previously described [12]. In order to confirm the effect of the main QTL on Chr 2 between *D2Mit62* and *D2Mit304* (the 129-derived locus for resistance to CH on Chr 2; *Lrch*), congenic strain mice in which the *Lrch* allele is introduced into the susceptible DW strain was generated by a marker-assisted speed congenic strategy [18]. In brief, among the male mice harboring the 129 alleles between *D2Mit62* and *D2Mit304*, the male mice with the highest percentage of the DW genetic background, evaluated using 72 markers (Table S1), were selected for breeding the next generation. Backcrossing was repeated six times and finally, congenic mice possessing heterozygous 129 alleles between *D2Mit62* and *D2Mit304* (DW.129-[*D2Mit62-D2Mit304*]; CG) were produced. Female CG mice heterozygous for both *Tpst2^grt^* and *Lrch* alleles were then crossed with DW male mice heterozygous for *Tpst2^grt^* and their progenies were used for congenic strain analysis. The animal room was air-conditioned at 22±4°C, maintained at 40–60% relative humidity, and mice were maintained under a 12 hr light-dark cycle. A standard laboratory diet, CE-2 (Nihon Clea, Tokyo, Japan), and tap water were available *ad libitum*.

### Genotyping Analysis

We used 49 (male = 26, female = 23) BC-*Tpst2^grt^* mice for a genome-wide scan. Extraction of genomic DNA from tail clips was performed by a standard method. For QTL analysis, a total of 74–75 informative microsatellite markers spanning 19 autosomes were used for the genotyping analysis, as listed in Table S1. The map positions of the microsatellite markers were based on information from the Mouse Genome Informatics of Jackson Laboratory (MGI; http://www.informatics.jax.org/, MGI_4.41). PCR was carried out with a cycling sequence of 95°C for 5 min (one cycle), followed by 35 cycles consisting of denaturation at 95°C for 30 sec, primer annealing at 55°C for 30 sec, and extension at 72°C for 30 sec. Amplified samples were electrophoresed with 10% polyacrylamide gels and stained with ethidium bromide. The stained gels were then visualized and photographed under an ultraviolet lamp.

### QTL Analysis

For the evaluation of resistance to TD-associated CH induced by *Tpst2^grt^*, we measured body weights at 5, 8 and 10 weeks of age and conducted principal component analysis. The first principal component, which provides the comprehensive value of body weights at each time-point and reflects a synthetic view of growth retardation, was used as quantitative trait for CH. Principal component analysis was performed with free software program for multivariate analysis (mulvar95; http://www.vector.co.jp/soft/win95/edu/se203904.html). QTL analysis was performed with the Map Manager QTXb20, software program that uses a maximum likelihood algorithm with “interval mapping” and “simultaneous search”, and permits better localization of loci and exclusion mapping [19]. Recombination frequencies (%) were converted into genetic distance (cM) using the Kosambi map function. This program provides linkage data as a likelihood ratio statistic (LRS) score. The LRS values were calculated by 10000 random permutations of the trait values relative to genotypes of the marker loci. Genome-wide significance thresholds were set at the 37^th^ (“suggestive”), 95^th^ (“significant”), and 99.9^th^ (“highly significant”) percentiles, which correspond to genome-wide α values of 0.63, 0.05 and 0.001 times, respectively, based on 10000 random permutation [20]. Since there are significant sex differences in weekly body weight, the principal component analysis and QTL analysis were performed separately for each sex.

### Histological Analysis of Thyroid Glands

Thyroid glands from 10-week-old mice were fixed with 5% neutral-buffered formalin and embedded in paraffin. Serial sections of thyroids were cut at 3 µm and stained with hematoxylin-eosin. Thyroid indices were calculated as previously reported [17]. In brief, the thyroid index indicates the average ratio of total colloid areas per thyroid cross-section area.

### Statistics

Statistical analyses were performed using the Stat-View program (SAS Institute Inc. Cary, USA). T-tests were used to compare the differences in body weight and thyroid index between each genotype. The correlation between body weight and thyroid index was analyzed using Pearson's correlation test. Scheffé's F tests were conducted for multiple comparisons between the body weights of each genotype in the congenic strain analysis. Statistical significance was set at *P*<0.05.

## Results

### Phenotypic Characterization of BC-*Tpst2^grt^* Mice

**Figure 1:**
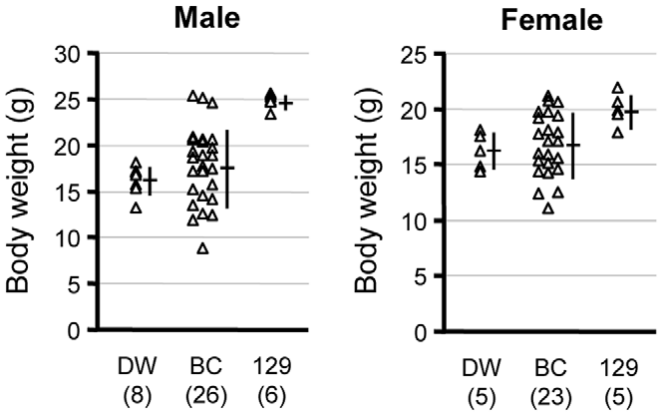
Plots of the individual body weights of DW-*Tpst2^grt^*, BC-*Tpst2^grt^* and 129-*Tpst2^grt^* mice at 8 weeks of age. Individual numbers are given in parentheses. Mean value and the standard deviation (s.d.) are indicated by horizontal and vertical lines, respectively.

To verify the effect of genetic background on susceptibility to growth retardation induced by *Tpst2^grt^*, the body weight of forty-nine (male = 26, female = 23) BC-*Tpst2^grt^* mice was measured at 5, 8 and 10 weeks of age. Figure 1 shows the plots of body weights of BC-*Tpst2^grt^* mice and mice homozygous for *Tpst2^grt^* in DW and 129 congenic strains (DW-*Tpst2^grt^* and 129-*Tpst2^grt^* mice, respectively) at 8 weeks of age [17]. The body weight of each individual BC-*Tpst2^grt^* mouse of either sex varied compared to that of DW-*Tpst2^grt^* or 129-*Tpst2^grt^* mice; i.e., some mice showed severe growth retardation similar to DW-*Tpst2^grt^* mice whereas, other mice showed growth comparable to that of resistant 129-*Tpst2^grt^* mice. This scattered distribution was also observed at 5 and 10 weeks of age (data not shown). Complete recovery from dwarfism observed in some backcross progenies of both sexes suggests that the tolerant gene(s) to growth retardation by CH in the 129 strain follows the autosomal dominant inheritance pattern. Also, the scattered distribution of body weight in BC-*Tpst2^grt^* mice suggests that several genes are involved in the resistance.

### Genome-wide Linkage Analysis for Mapping of Tolerant Genes for CH-related Growth Retardation in Backcross Mice

**Figure 2:**
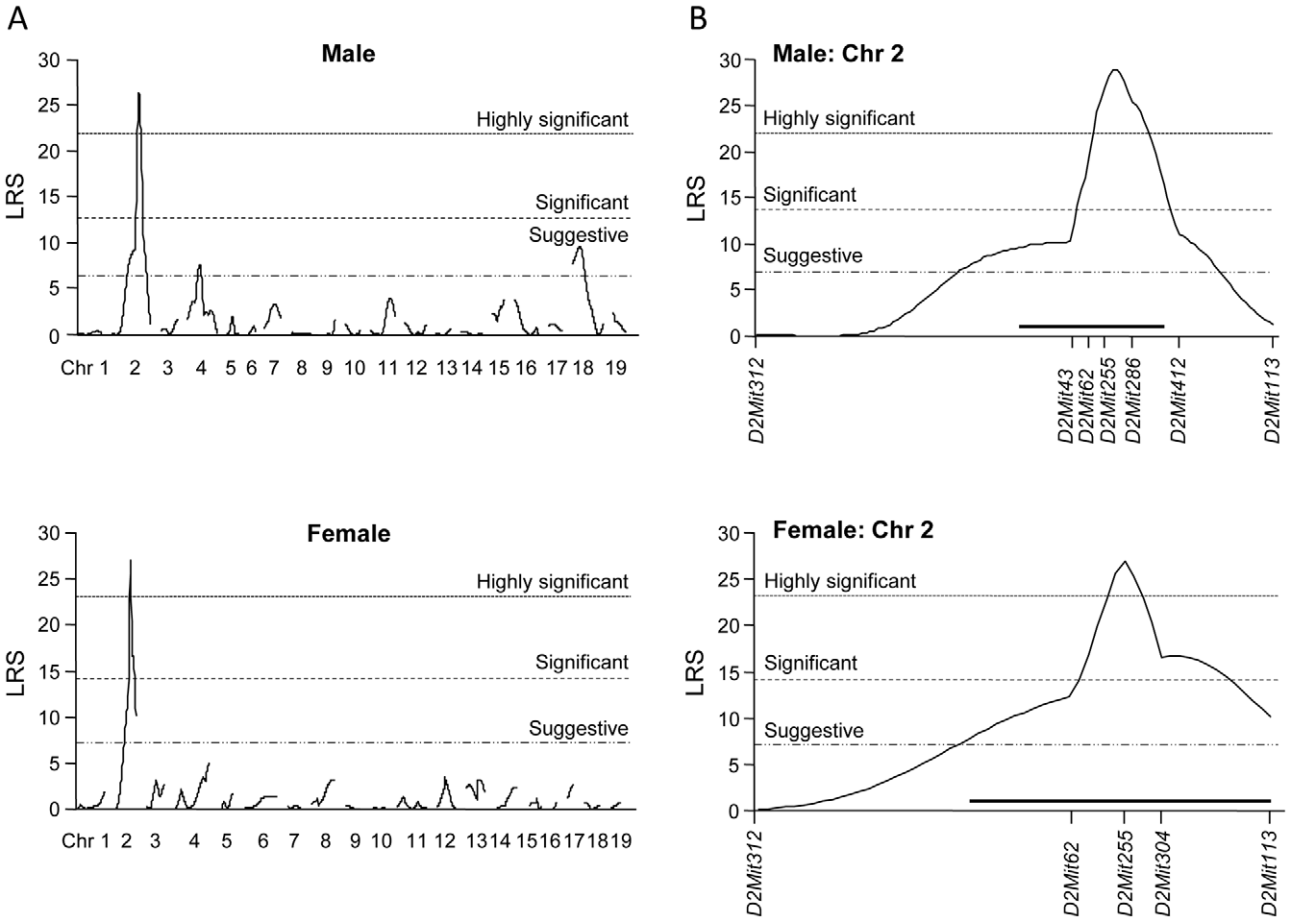
Linkage maps displaying the QTL using the first principal component as an indicator for CH. A) Genome-wide linkage maps. B) Details of highly significant linkages on Chr 2. Suggestive, significant and highly significant values are 6.9, 13.7 and 21.8 for male, 7.0, 14.1 and 23.0 for female, respectively. The maximum LRS score on Chr 2 is 28.9 for male and 27.0 for female, respectively. Horizontal black bars in Fig. 2B indicate 95% confidence intervals.

To identify the modifier genes for CH, we carried out a genome-wide linkage analysis using BC-*Tpst2^grt^* mice. The first principal component calculated from body weights at 5, 8 and 10 weeks of age was used as an indicator for CH. The genome-wide and detailed linkage maps on Chr 2 are shown in Fig. 2. Although there were suggestive QTLs on Chrs 4 and 18 in males (detailed maps are shown in Fig. S1), highly significant linkages were observed on Chr 2 in both sexes. Table 1 summarizes the microsatellite markers linked to the trait with highest LRS value on each Chr, percentage of the variance, genome-wide *P* value and phenotypic values of each genotype. All QTL alleles increased resistance to growth retardation by *Tpst2^grt^* when contributed by the 129 strain. Since high percentages of the variance (63–69%) supported the validity of the QTL on Chr 2, as a powerful modifier for growth delay induced by the *Tpst2^grt^* mutation, we focused QTL on Chr 2 exclusively. Integrating the results for both sexes (Fig. 2B), the main modifier locus is estimated to be between *D2Mit62* and *D2Mit304*, and particularly close to *D2Mit255*.

**Table 1:** Characteristics of QTLs for growth retardation.

Sex	Marker	LRS	%	*P*	Homozygous	Heterozygous
Male	*D2mit255*	25.5	63	<10^−5^	16.20±2.92	23.44±2.60*
	*D4mit152*	8.4	28	0.0037	17.64±3.96	22.13±4.21*
	*D18mit110*	8.5	29	0.0036	16.80±4.98	21.02±3.85*
Female	*D2mit255*	27.0	69	<10^−5^	15.73±1.95	20.70±1.97*

The microsatellite markers linked to the indicator for CH with the highest LRS value on each Chr, percentage of the variance, genome-wide *P* value detected by marker regression analysis based on 10000 permutation replicates and body weights of each genotype at 10 weeks age are indicated. %: percentage of the variance. *P*: genome-wide *P* value as calculated by QTX software. Means ± s.d. are shown.

*: *P*<0.01.

### Histological Evaluation of the Thyroid Glands of BC-*Tpst2^grt^* Mice

**Figure 3:**
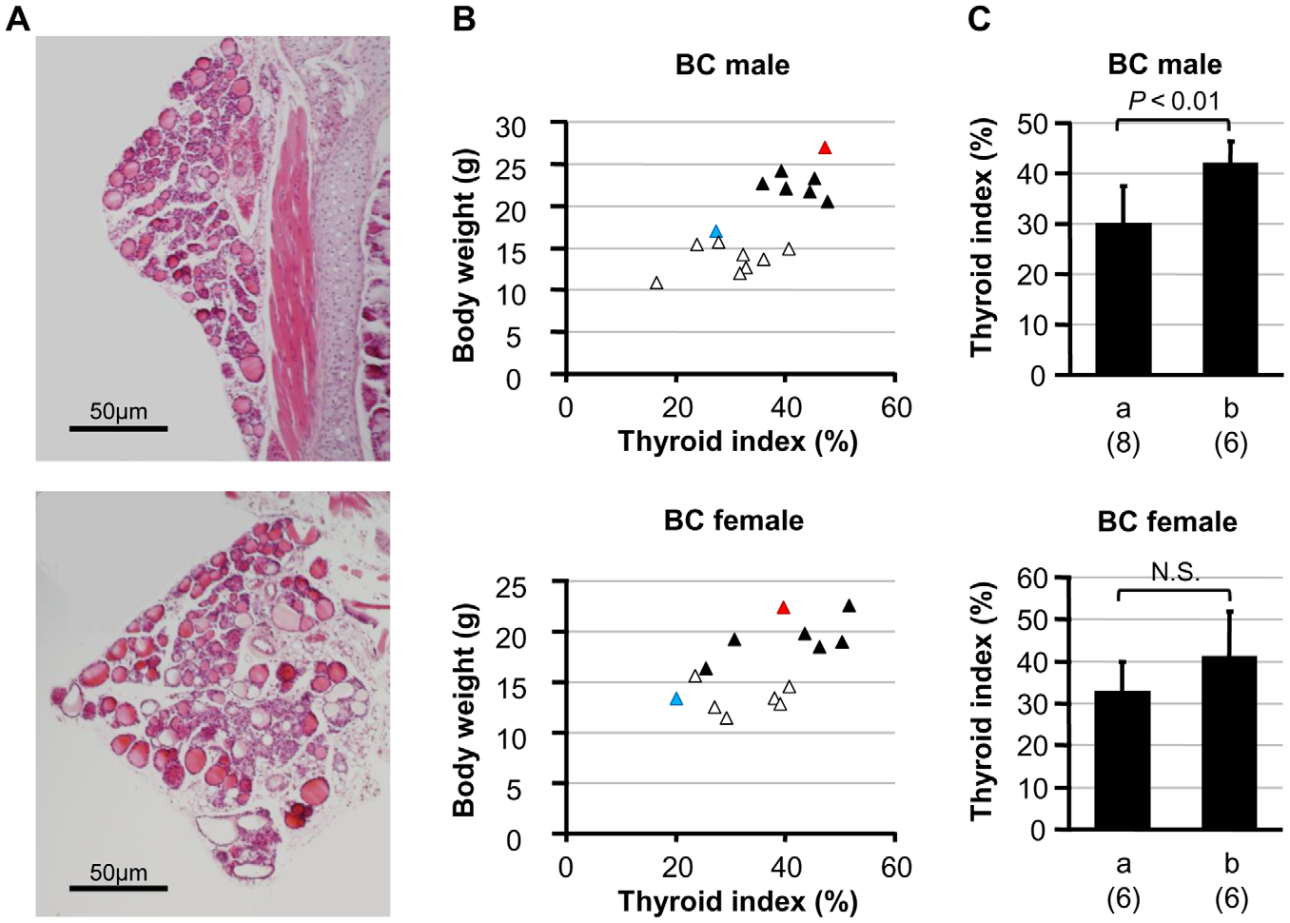
The severity of growth retardation in BC-*Tpst2^grt^* mice is related to thyroid hypoplasia. A) Representative cross-sections of thyroid glands of BC-*Tpst2^grt^* mice at 10 weeks of age. Upper: a male BC-*Tpst2^grt^* mouse with severe growth retardation (body weight was 35.2% that of the littermate control; thyroid index = 16.3%). Lower: a male BC-*Tpst2^grt^* mouse with mild growth retardation (body weight was 81.4% that of the littermate control; thyroid index = 47.4%). B) Plots of body weight and thyroid index of BC-*Tpst2^grt^* at 10 weeks of age. Open triangles: BC-*Tpst2^grt^* homozygous for *D2Mit255* (n = 8 and 6 for male and female, respectively), solid triangles: BC-*Tpst2^grt^* heterozygous for *D2Mit255* (n = 6 and 6 for male and female, respectively), blue triangles: the average of DW-*Tpst2^grt^* (n = 4 and 3 for male and female, respectively), red triangles: the average of 129-*Tpst2^grt^* (n = 3 and 4 for male and female, respectively). C) Comparison of thyroid indices between genotypes at *D2Mit255* in BC-*Tpst2^grt^* mice. a: homozygous, b: heterozygous for *D2Mit255*, respectively. Individual numbers are given in parentheses. Vertical lines indicate s.d. N.S.: not significant.

Since the tolerance to growth retardation in 129-*Tpst2^grt^* mice is due to their normal thyroid development and function in spite of hyporeactivity to TSH signaling [17], we conducted a histological analysis of the thyroid glands. Thyroid sections of randomly selected 10-week-old BC-*Tpst2^grt^* mice showed some correlation between the severity of growth retardation and the degree of thyroid hypoplasia (Fig. 3A). Namely, the thyroid glands of BC-*Tpst2^grt^* mice with severe growth delay show more severe hypoplastic symptoms, such as a decrease in the number of colloid-filled follicles, diversity in follicle size and increased replacement of parenchyma cells with adipocytes, compared to those with milder growth retardation. Next, we carried out a quantitative evaluation of the severity of thyroid dysplasia using a “thyroid index” (average ratio of total colloid areas per thyroid cross-section area) as previously described [17]. There was no correlation between body weight and thyroid index in either sexes BC-WT (Fig. S2: *P* = 0.1495, *r* = 0.394 and n = 15 in males; *P* = 0.9687, *r* = −0.015, n = 10 in females), although there were significant correlations between them in BC-*Tpst2^grt^* mice of both sexes (Fig. 3B: *P* = 0.0042, *r* = 0.698 and n = 14 in males; *P* = 0.0441, *r* = 0.586 and n = 12 in females). This result demonstrates that the growth retardation in BC-*Tpst2^grt^* mice is correlated to the severity of thyroid hypoplasia. In addition, although there was no significant difference in females, male BC-*Tpst2^grt^* mice heterozygous for *D2Mit255* showed a significant increase in thyroid index compared to male BC-*Tpst2^grt^* mice homozygous for *D2Mit255* (Fig. 3C). There was no significant difference when male BC-*Tpst2^grt^* mice were grouped according to genotype at the weaker QTLs on Chrs 4 and 18, respectively (data not shown). These results suggest that the main CH resistant gene(s) is located on Chr 2 near *D2Mit255*, and that it is involved in thyroid development.

### Evaluation of the Effects of *Lrch* by Analysis of Congenic Mice

**Figure 4:**
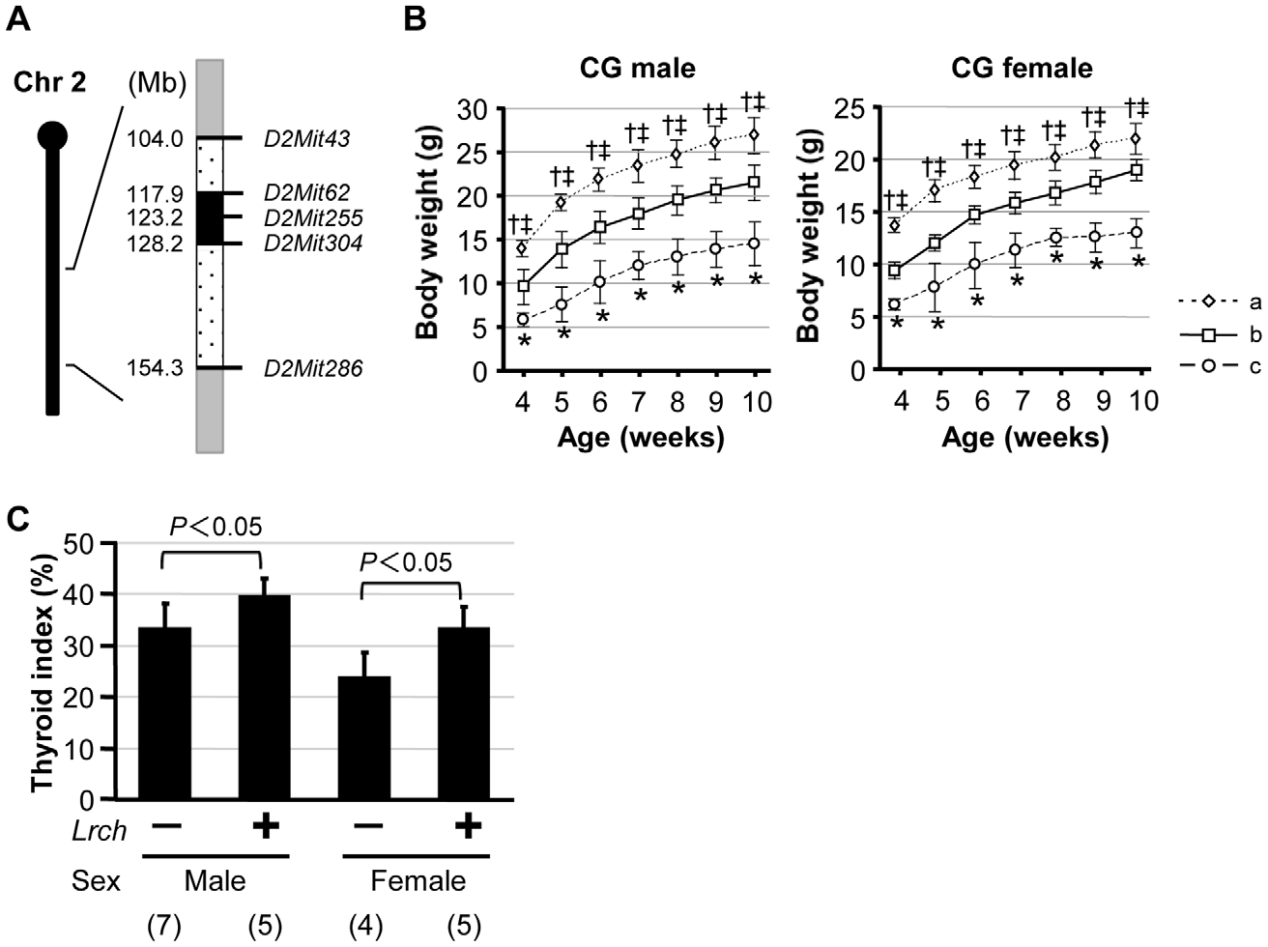
Evaluation of the effect of *Lrch* on TD-associated growth retardation by congenic strain analysis. A) Schematic diagram of the genomic structure surrounding *Lrch* in CG mice. Gray bars indicate the minimum genomic regions derived from DW. A black bar indicates a genomic region into which 129-derived alleles were introduced. Dotted bars represent recombined regions between the DW and 129 genomes. The numbers to the left of the bars represent physical locations based on the MGI. B) Growth curves of CG mice. a: littermate controls, b: CG-*Tpst2^grt^* mice heterozygous for the 129-derived *Lrch* allele, c: CG-*Tpst2^grt^* mice without the 129-derived *Lrch* allele. Individual numbers of a, b, c are 5, 4 and 5 for males, and 9, 5 and 2 for females, respectively. Vertical lines indicate s.d. *P*<0.05: *; b versus c, †; b versus a, ‡; c versus a. C) Thyroid indices of CG-*Tpst2^grt^*. Individual numbers are given in parentheses.

In order to validate the effects of the 129-derived resistant locus (locus for resistance to CH: designated *Lrch*), a DW congenic mouse strain carrying 129 alleles at this locus was generated (Fig. 4A). Figure 4B shows the growth curves of each genotype of CG mice. CG mice with wild-type *Tpst2* alleles were used as littermate controls. Among the CG mice homozygous for *Tpst2^grt^* (CG-*Tpst2^grt^*), mice with the 129-derived *Lrch* allele showed partial recovery from growth retardation in both sexes, compared to mice without the 129-derived *Lrch* allele. The *Lrch* allele showed no obvious influence on growth in littermate controls (data not shown). In addition, thyroid index of CG-*Tpst2^grt^* with the 129-derived *Lrch* allele showed a significant elevation compared to CG-*Tpst2^grt^* without the 129-derived *Lrch* allele for both sexes (Fig. 4C). Thus, the congenic analysis confirmed the significant impact of *Lrch*, located between *D2Mit43* and *D2Mit286*, on TD-associated growth retardation.

## Discussion

In a previous study, we demonstrated that growth retardation by *Tpst2^grt^*-related CH is influenced by genetic background. Mice harboring homozygotic *Tpst2^grt^* in a B6 genetic background, as well as DW-*Tpst2^grt^*, show susceptibility to CH. In contrast, the thyroid function in 129-*Tpst2^grt^* mice is fully recovered irrespective of TPST2 deficiency, and the 129 strain is remarkably resistant to growth retardation by CH [17]. Most gene targeting is carried out in cultured ES cells derived from the 129 strain because of its higher efficiency of germline transmission. Our data indicated that thyroid-related gene knockout mice might sometimes appear phenotypically normal in a 129 genetic background.

In this study, to identify the resistant gene(s) for CH, we performed QTL analysis using backcross progenies from susceptible DW and resistant 129 strain mice. Although the mapping resolution is not good enough to distinguish two QTLs close to each other, QTL analysis using the first principal component calculated from pubertal body weights identified a highly significant QTL on Chr 2 adjacent to *D2Mit255*, and suggestive QTLs on Chrs 4 and 18 (Fig. 2). In fact, 129-derived alleles of each QTL increased resistance to dwarfism in both BC-*Tpst2^grt^* and CG-*Tpst2^grt^* mice (Table 1 and Fig. 4B). Although body weight is a compound trait reflecting the weights of lean muscle and bones as well as fat mass, a significant correlation between the severity of growth delay and the degree of thyroid hypoplasia suggested the involvement of thyroid dysplasia to growth retardation in BC-*Tpst2^grt^* mice (Fig. 3A and B). Indeed, a significant elevation in thyroid index for both sexes was observed in CG-*Tpst2^grt^* with the 129-derived *Lrch* allele, compared to CG-*Tpst2^grt^* without the 129-derived *Lrch* allele (Fig. 4C). This result confirmed the significant impact of *Lrch*, located between *D2Mit43* and *D2Mit286*, on TD-associated growth retardation. Since there were complete recovery effects on dwarfism and thyroid development in backcross population (Fig. 1A and 3B), and the resistant gene(s) to CH in 129 strain is supposed to follow dominant inheritance, we used backcross population for examination of growth and thyroid development in CG mice. The partial recovery from growth retardation in CG-*Tpst2^grt^* mice with the 129-derived *Lrch* (Fig. 4B; compared to littermate controls) suggests the effects of other QTLs, though dosage effects of the *Lrch* cannot be excluded. Further examination using intercross population will prove this problem.

Besides the major QTL on Chr 2, we detected male specific QTLs on Chrs 4 and 18. This may explain the sex bias for TD in human. In fact, gender bias in TD is reported in humans, with a female: male ratio of 1.4 for entire TD, 1.9 for ectopic thyroid glands and 0.9 for athyreosis [5], although the reason for this remains unclear. It is possible that the TD resistant genes are influenced by the male sex hormone. Alternatively, QTLs detected in this study might be correlated with genes differentially expressed in a gender-specific manner.

By use of CH mice with TD caused by a double-heterozygous null mutation in genes encoding *Ttf1* and *Pax8* (DHTP), Amendola *et al*. also found that the B6 strain has a greater susceptibility to CH in comparison to 129 strain mice [10]. They mapped modifier genes for CH on Chrs 2 and 5 by genetic linkage analysis, and further localized the CH-related QTL on Chr 2 (*HTRC2*) from 112–121 Mb [21]. In the present study, we localized the major modifier QTL on Chr 2 from 117.9–123.2 Mb by QTL mapping and confirmed that *Lrch* is located between 104.0–154.3 Mb by congenic strain analysis. This locus overlaps *HTRC2*. From a subsequent transcriptional assay and sequencing of candidate genes, they concluded that the amino acid substitution of highly conserved tyrosine 273 to phenylalanine in *Dnajc17* (119.0 Mb), encoding for a member of the type III heat-shock protein-40 family, results in reduced thyroglobulin (*Tg*) transcription, leading to CH in B6 mice [21]. We also found a Y273F polymorphism in *Dnajc17* in DW strain mice, which is the same as the susceptible B6 mice in the above study. However, we could not detect any deficiency in *Tg* transcription, synthesis, transportation or secretion in DW-*Tpst2^grt^* mice [12], [22]. As the pathology of CH differs between each model, modifier genes for *Tpst2^grt^*-induced CH may differ from those for DHTP-induced CH. The fact that the peak LRS on Chr 2 is between *D2Mit255* and *D2Mit304* (123.2–128.2 Mb), rather than between *D2Mit62* and *D2Mit255* (117.9–123.2 Mb) in male (Fig. 2) supports the above idea.

Since the *Tpst2^grt^* mutation causes TSH hyporesponsiveness [12], we predict that the resistant factor(s) for CH in the 129 strain are mainly involved in signal transduction downstream of TSHR or TSH-independent activity in thyrocyte proliferation and thyroid hormone biosynthesis. The positional MEDLINE (http://omicspace.riken.jp/PosMed/) revealed that there are at least 18 genes related to thyroid development and function between *D2Mit62* and *D2Mit304* (Table 2). In particular, dual oxidase 2 (*Duox2*) (122.1 Mb), dual oxidase maturation factor 2 (*Duoxa2*) (122.1 Mb) and Kv channel interacting protein 3 (*Kcnip3*, also known as *Dream*) (127.3 Mb) agree with our prediction. *Duox2* and *Duoxa2* attracted our interest in that these genes are involved in several forms of CH in human and mice [23], [24]. DUOX2 is one of the NADPH oxidases producing H_2_O_2_, which is required for iodination of TG whose modification is prerequisite for thyroid hormone synthesis. In addition to its transcription, enzymatic activity of DUOX2 is also regulated by TSH signaling in human [25], [26]. DUOXA2 is essential for DUOX2 maturation and trafficking from the endoplasmic reticulum to the plasma membrane where DUOX2 functions [25], [27]. KCNIP3 is preferentially expressed in the central nervous system and thyroid gland in mice, and binds to the downstream regulatory element where its binding to the sequence suppresses the transcription of target genes [28]. Recently, transcriptional regulation of thyroid specific genes (for example, *Tg*, *Pax8* and *Foxe1*) by KCNIP3 was reported [29], [30]. Furthermore, constitutively active mutant KCNIP3 interferes with thyroid cell proliferation *in vitro* [30] and induces thyroid enlargement and nodular development in mice [31]. Moreover, the regulation of TSHR activity by KCNIP3 is suggested in thyrocytes [31]. We examined the expression of these possible candidate genes (*Dnajc17*, *Duox2*, *Duoxa2* and *Kcnip3*) in thyroid glands of DW and 129 strain mice with semi-quantitative RT-PCR. However, there was no quantitative difference observed (data not shown). To determine the resistant genes, the generation and analysis of subcongenic mice together with further genetic study is required.

**Table 2:** Candidate genes for *Lrch* between *D2Mit62* and *D2Mit304*.

Gene Symbol	Gene Description	Physical Position (Mb)
***D2Mit62***		117.9
*Thbs1*	Thrombospondin 1	117.9
*Bmf*	BCL2 modifying factor	118.4
*Plcb2*	Phospholipase C, beta 2	118.5
*Dnajc17*	DnaJ (Hsp40) homolog, subfamily C, member 17	119.0
*Spint1*	Serine protease inhibitor, Kunitz type 1	119.1
*RhoV*	Ras homolog gene family, member V	119.1
*Iptka*	Inositol 1,4,5-triphosphate 3-kinase A	119.6
*Mga*	MAX gene associated	119.7
*Ubr1*	Ubiquitin protein ligase E3 component n-recognition 1	120.7
*Duox2*	Dual oxidase 2	122.1
*Duoxa2*	Dual oxidase maturation factor 2	122.1
***D2Mit255***		123.2
*Fgf7*	Fibroblast growth factor 7	125.9
*Usp8*	Ubiquitin specific peptidase 8	126.5
*Ciao1*	Cytosolic iron-sulfur protein assembly 1 homolog (S. cerevisiae)	127.1
*Dusp2*	Dual specificity phosphatase 2	127.2
*Adra2b*	Adrenergic receptor, alpha 2b	127.2
*Kcnip3*	Kv channel interacting protein 3	127.3
*Bcl2l11*	BCL2-like 11	128.0
***D2Mit304***		128.2

In conclusion, genome-wide analysis and a congenic mouse study identified a major modifier gene for *Tpst2^grt^*-induced CH on Chr 2, between 104.0–154.3 Mb and adjacent to *D2Mit255*. The major QTL identified in our study overlaps with *HTRC2* which was identified in another study. It is intriguing that genetic linkage analyses using distinct CH models with different origins mapped the major QTL on the same region on Chr 2. Identification of genes(s) responsible for resistance to hypothyroidism in 129-*Tpst2^grt^* mice will provide insights into the molecular events involved in thyroid development. Moreover, the discovery of novel causative gene(s) should lead to the development of treatment strategies for thyroid cell disorders including thyroid tumors and hyperthyroidism, as well as hypothyroidism.

## Supporting Information

Figure S1 **Details of male-specific suggestive linkages on Chr 4 and Chr 18.** Suggestive, significant and highly significant values are 6.9, 13.7 and 21.8, respectively. Horizontal black bars represent 95% confidence intervals. The maximum LRS is 8.4 for Chr 4, and 10.6 for Chr 18.
(TIF)

Figure S2 **Plots of body weight and thyroid index of BC-WT mice at 10 weeks of age.** Solid triangles: BC-WT (n = 15 and 10 for male and female, respectively), blue triangles: the average of the DW mice with wild-type *Tpst2^grt^* allele (n = 5 and 3 for male and female, respectively), red triangles: the average of the 129 mice with wild-type *Tpst2^grt^* allele (n = 5 and 3 for male and female, respectively).
(TIF)

Table S1 List of microsatellite markers used for the QTL analysis.
(XLSX)

## References

[1] Van Vliet G (2003). Development of the thyroid gland: lessons from congenitally hypothyroid mice and men.. Clinical Genetics.

[2] Kopp P (2002). Perspective: genetic defects in the etiology of congenital hypothyroidism.. Endocrinology.

[3] Léger J, Marinovic D, Garel C, Bonaïti-Pellié C, Polak M (2002). Thyroid developmental anomalies in first degree relatives of children with congenital hypothyroidism.. J Clin Endocrinol Metab.

[4] Hashemipour M, Hasani N, Amini M, Heidari K, Sajadi A (2010). Thyroid function abnormalities among first-degree relatives of Iranian congenital hypothyroidism neonates.. Pediatr Int.

[5] Castanet M, Marinovic D, Polak M, Léger J (2010). Epidemiology of thyroid dysgenesis: the familial component.. Horm Res Paediatr.

[6] Fagman H, Nilsson M (2011). Morphogenetics of early thyroid development.. J Mol Endocrinol.

[7] Dentice M, Cordeddu V, Rosica A, Ferrara AM, Santarpia L (2006). Missense mutation in the transcription factor NKX2-5: a novel molecular event in the pathogenesis of thyroid dysgenesis.. J Clin Endocrinol Metab.

[8] Fagman H, Nilsson M (2010). Morphogenesis of the thyroid gland.. Mol Cell Endocrinol.

[9] Castanet M, Sura-Trueba S, Chauty A, Carré A, de Roux N (2005). Linkage and mutational analysis of familial thyroid dysgenesis demonstrate genetic heterogeneity implicating novel genes.. Eur J Hum Genet.

[10] Amendola E, De Luca P, Macchia PE, Terracciano D, Rosica A (2005). A mouse model demonstrates a multigenic origin of congenital hypothyroidism.. Endocrinology.

[11] Tomita K, Yoshida T, Morita J, Atsumi S, Totsuka T (1995). *In vivo* responsiveness of thyroid glands to TSH in normal and novel ‘growth-retarded’ mice: transient elevation in normal mice and impairment in ‘growth-retarded’ mice.. J Endocrinol.

[12] Sasaki N, Hosoda Y, Nagata A, Ding M, Cheng JM (2007). A mutation in *Tpst2* encoding tyrosylprotein sulfotransferase causes dwarfism associated with hypothyroidism.. Mol Endocrinol.

[13] Costagliola S, Panneels V, Bonomi M, Koch J, Many MC (2002). Tyrosine sulfation is required for agonist recognition by glycoprotein hormone receptors.. EMBO Journal.

[14] Bonomi M, Busnelli M, Persani L, Vassart G, Costagliola S (2006). Structural differences in the hinge region of the glycoprotein hormone receptors: evidence from the sulfated tyrosine residues.. Mol Endocrinol.

[15] Postiglione MP, Parlato R, Rodriguez-Mallon A, Rosica A, Mithbaokar P (2002). Role of the thyroid-stimulating hormone receptor signaling in development and differentiation of the thyroid gland.. P Natl Acad Sci U S A.

[16] Persani L, Calebiro D, Cordella D, Weber G, Gelmini G (2010). Genetics and phenomics of hypothyroidism due to TSH resistance.. Mol Cell Endocrinol.

[17] Hosoda Y, Sasaki N, Agui T (2010). Hypothyroid phenotype of the *Tpst2* mutant mouse is dependent upon genetic background.. Biomed Res.

[18] Markel P, Shu P, Ebeling C, Carlson GA, Nagle DL (1997). Theoretical and empirical issues for marker-assisted breeding of congenic mouse strains.. Nat Genet.

[19] Manly KF, Cudmore RH, Meer JM (2001). Map Manager QTX, cross-platform software for genetic mapping.. Mamm Genome.

[20] Lander E, Kruglyak L (1995). Genetic dissection of complex traits: guidelines for interpreting and reporting linkage results.. Nat Genet.

[21] Amendola E, Sanges R, Galvan A, Dathan N, Manenti G (2010). A locus on mouse chromosome 2 is involved in susceptibility to congenital hypothyroidism and contains an essential gene expressed in thyroid.. Endocrinology.

[22] Cheng JM, Ding M, Miyamoto T, Fujimori O, Agui T (2002). Investigation of post-transcriptional events of the thyroglobulin in the thyroid gland of the hypothyroid growth-retarded mouse DW/J-*grt*.. Nagoya Medical Journal.

[23] Grasberger H (2010). Defects of thyroidal hydrogen peroxide generation in congenital hypothyroidism.. Mol Cell Endocrinol.

[24] Johnson KR, Marden CC, Ward-Bailey P, Gagnon LH, Bronson RT (2007). Congenital hypothyroidism, dwarfism, and hearing impairment caused by a missense mutation in the mouse dual oxidase 2 gene, *Duox2*.. Mol Endocrinol.

[25] De Deken X, Wang D, Many MC, Costagliola S, Libert F (2000). Cloning of two human thyroid cDNAs encoding new members of the NADPH oxidase family.. J Biol Chem.

[26] Rigutto S, Hoste C, Grasberger H, Milenkovic M, Communi D (2009). Activation of dual oxidases Duox1 and Duox2: differential regulation mediated by cAMP-dependent protein kinase and protein kinase C-dependent phosphorylation.. J Biol Chem.

[27] Grasberger H, Refetoff S (2006). Identification of the maturation factor for dual oxidase. Evolution of an eukaryotic operon equivalent.. J Biol Chem.

[28] Carrión AM, Link WA, Ledo F, Mellström B, Naranjo JR (1999). DREAM is a Ca^2+^-regulated transcriptional repressor.. Nature.

[29] Rivas M, Mellström B, Naranjo JR, Santisteban P (2004). Transcriptional repressor DREAM interacts with thyroid transcription factor-1 and regulates thyroglobulin gene expression.. J Biol Chem.

[30] D'Andrea B, Di Palma T, Mascia A, Motti ML, Viglietto G (2005). The transcriptional repressor DREAM is involved in thyroid gene expression.. Exp Cell Res.

[31] Rivas M, Mellström B, Torres B, Cali G, Ferrara AM (2009). The DREAM protein is associated with thyroid enlargement and nodular development.. Mol Endocrinol.

