# Supplementary figures and images for: Identifying Quantitative Trait Loci Affecting Resistance to Congenital Hypothyroidism in 129+Ter/SvJcl Strain Mice
Source: PLoS One. 2012 Jan 27;7(1):e31035. doi: 10.1371/journal.pone.0031035 (PMC3267771; doi:10.1371/journal.pone.0031035)

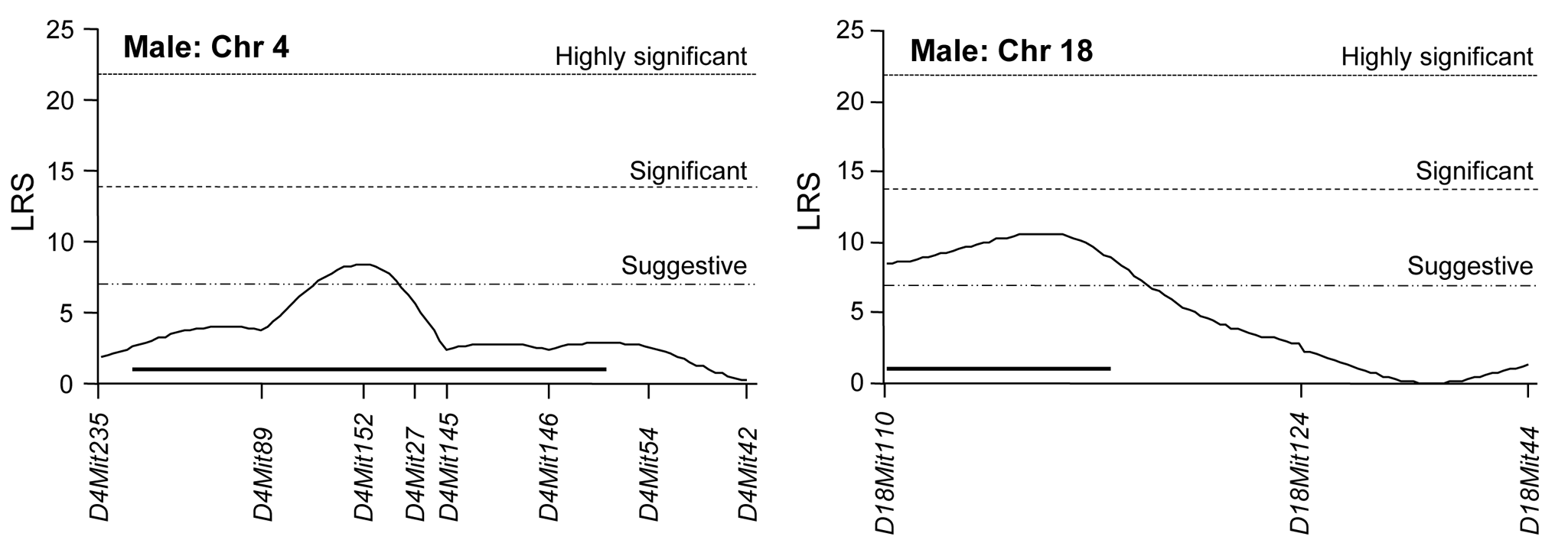

Supplement: Figure S1 — Details of male-specific suggestive linkages on Chr 4 and Chr 18. Suggestive, significant and highly significant values are 6.9, 13.7 and 21.8, respectively. Horizontal black bars represent 95% confidence intervals. The maximum LRS is 8.4 for Chr 4, and 10.6 for Chr 18. (TIF) [file pone.0031035.s001.tif]

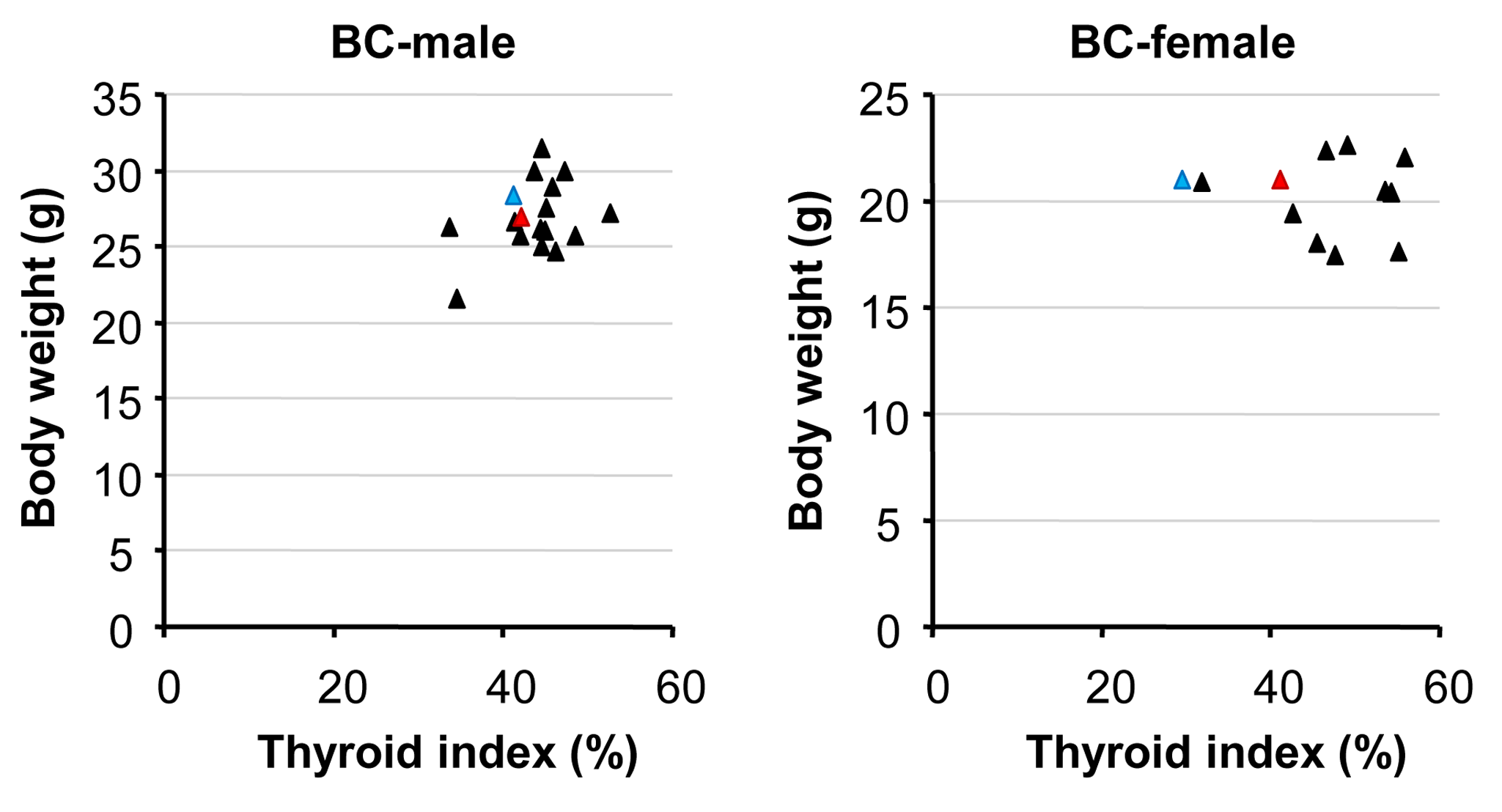

Supplement: Figure S2 — Plots of body weight and thyroid index of BC-WT mice at 10 weeks of age. Solid triangles: BC-WT (n = 15 and 10 for male and female, respectively), blue triangles: the average of the DW mice with wild-type Tpst2grt allele (n = 5 and 3 for male and female, respectively), red triangles: the average of the 129 mice with wild-type Tpst2grt allele (n = 5 and 3 for male and female, respectively). (TIF) [file pone.0031035.s002.tif]
